# Retraction: Tumors with TSC mutations are sensitive to CDK7 inhibition through NRF2 and glutathione depletion

**DOI:** 10.1084/jem.2019025105292026r

**Published:** 2026-06-12

**Authors:** Mahsa Zarei, Heng Du, Amin H. Nassar, Rachel E. Yan, Krinio Giannikou, Sneha H. Johnson, Hilaire C. Lam, Elizabeth P. Henske, Yubao Wang, Tinghu Zhang, John Asara, David J. Kwiatkowski

Vol. 216, No. 11 | https://doi.org/10.1084/jem.20190251 | September 10, 2019

This article has been retracted at the request of the authors, save for Sneha H. Johnson, who could not be reached due to outdated contact information. Following a joint institutional review by Harvard Medical School and Brigham and Women’s Hospital, Figs. 1 B, 2 D, 4 G, and 6 D have been found unreliable due to image duplication, and quantitative data in Fig. 6, A and C, do not accurately represent the source data. The authors have lost confidence in the data and believe the most responsible course of action is to retract the article.

The authors sincerely apologize for any adverse consequences that this might cause to the scientific community.

